# Highly flexible touch screen panel fabricated with silver-inserted transparent ITO triple-layer structures

**DOI:** 10.1039/c7ra13550e

**Published:** 2018-03-27

**Authors:** Chia-Ching Wu

**Affiliations:** Department of Applied Science, National Taitung University Taitung Taiwan Republic of China 9113718@gmail.com +886-089-517900 +886-089-518108

## Abstract

A flexible and transparent amorphous-indium tin oxide/silver/crystalline-indium tin oxide (a-ITO/Ag/c-ITO) triple-layer structure was prepared as an electrode for capacitive-type touch screen panels (TSPs). A very thin metal film of silver (Ag) was inserted between two ITO layers, and the triple-layer structures were deposited on a colorless polyimide (CPI) substrate by a sputtering method. It was found that the tunable electrical and optical properties of the a-ITO/Ag/c-ITO triple-layer structures were critically affected by the thickness of the inserted Ag layer. The optimized flexible a-ITO/Ag/c-ITO triple-layer structure has low sheet resistance, high optical transmittance, and low surface roughness. In addition, during the 30 000 bending cycles, the resistance change (Δ*R*) of the flexible a-ITO/Ag/c-ITO triple-layer structure was 4.12%. For environmental reliability, the Δ*R* values of the flexible a-ITO/Ag/c-ITO triple-layer structure were 2.86% and 0.96% at the environmental temperature of 80 °C-50% and −40 °C, respectively. The above results indicate that the a-ITO/Ag/c-ITO triple-layer structure can be utilized to construct a promising TCO electrode. Finally, flexible and foldable capacitive-type TSPs were fabricated with multiple touch points using the a-ITO/Ag/c-ITO triple-layer electrode.

## Abstract 2

A flexible and transparent amorphous-indium tin oxide/silver/crystalline-indium tin oxide (a-ITO/Ag/c-ITO) triple-layer structure was prepared as an electrode for capacitive-type touch screen panels (TSPs).

## Graphical abstract

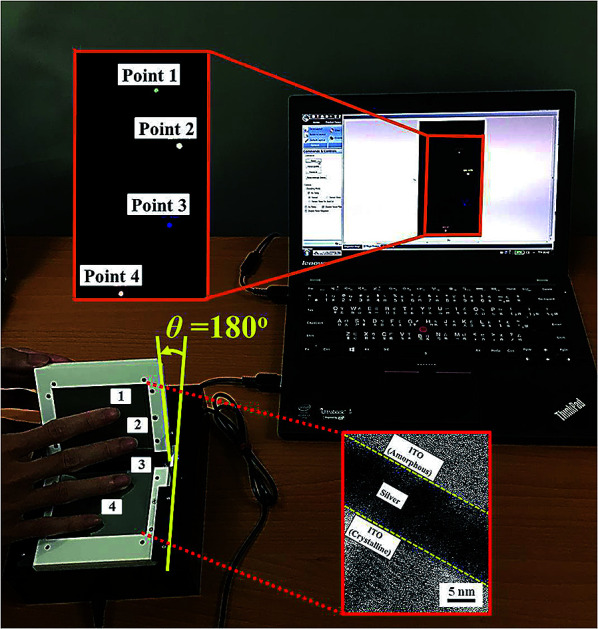

## 1. Introduction

Transparent conducting oxide (TCO) is essential for photosensitive electronic devices such as thin film solar cells,^1^ flat panel displays,^2^ touch screens,^3^ and organic light-emitting diodes (OLEDs).^4^ There are at least two important factors, including the resistivity and transmittance, that are used to evaluate the performance of a TCO thin film. Low sheet resistance and high transmittance are required especially when TCO is used as an electrode material. Due to the great advance of experimental exploration, a large variety of TCO thin films, including traditional metal oxides,^5,6^ graphene,^7^ carbon nanotubes,^8^ and metal nanostructures,^9–15^ have been successfully fabricated and widely applied in optoelectronic devices. Indium tin oxide (ITO) is the best choice for transparent conducting (TCO) films because of its low resistivity (<10^−4^ Ω cm) and high transmittance (>90%).

Touch screen panels (TSPs) have been considered as one of the key components in mobile communication devices. The capacitive-type TSPs are now being used instead of conventional resistive-type TSPs due to their capacity for multi-touch function and multitasking. Recently, flexible capacitive-type TSPs have been extensively investigated for application in flexible OLED displays. In these, the TCO electrode plays an important role in determining the performance of the flexible TSPs. The flexible TCO electrode should have mechanical robustness against substrate bending without resultant changes in its optical and electrical properties. In previous research, amorphous ITO thin film were widely employed as a flexible TCO material in flexible optoelectronic devices due to their high conductivity and transparency in the visible spectral range.^16^ However, cracks that easily form in the brittle amorphous ITO films have been considered as a critical drawback in flexible devices.^17^ In addition, crystallized ITO films showed a higher transmittance and lower resistivity than amorphous films.^18,19^

In order to eliminate the critical drawbacks of the ITO films, ITO sandwiching of thin metal films has been extensively investigated.^20,21^ Compared with single-layer ITO films, the ITO/metal/ITO triple-layer structures can effectively suppress the reflection from the metal layer in the visible range and yield better electrical conductivity, and therefore, this area of research has generated interest. The metal layer of TCO/metal/TCO multilayer structures is often comprised of gold (Au), silver (Ag), copper (Cu), or molybdenum (Mo). Triple-layer structures such as ITO/metal/ITO,^22,23^ ZnO/Cu/ZnO,^24^ IZO/Au/IZO,^25^ ZnO/Ag/ZnO,^26^ AZO/Ag/AZO,^27^ NTO/Ag/NTO,^28^ and ZTO/Ag/ZTO^29^ have been reported.

In this research, we studied the structural, electrical, and optical properties of the ITO/metal/ITO triple-layer structures, which were sputtered on a colorless polyimide (CPI) substrate. The transmittance, coefficient of thermal expansion, and hardness of the CPI were outstanding for plastic material as compared to that of polyimide (PI), polyethylene terephthalate (PET), polyethylene naphthalate (PEN), and polyester (PES). The effects of the thin metal film thickness of the ITO/metal/ITO triple-layer structures were investigated. Under optimized conditions, we fabricated a flexible capacitive-type TSP using the ITO/metal/ITO triple-layer structure. The capacitive-type TSPs have been substituted for conventional resistive-type TSPs due to their capacity for multi-touch function and multi-tasking. The multi-touch function of TSPs is critically dependent on the resistance and optical transparency of the touch sensor electrodes. Thus, this study can provide a practical solution to the existing problems characterizing flexible TCO materials and their TSP application.

## 2. Experimental detail

### 2.1 Fabrication of the ITO/Ag/ITO triple-layer structures

In previous reports, the higher transmittance of the TCO-Ag-TCO structure compared with that of the TCO-Au-TCO structure was explained by Ag having a lower absorption than Au in the visible region of 400–700 nm (5% *versus* 8%).^30–32^ In this investigation, a thin metal film of Ag was inserted between two flexible ITO thin films to form a triple-layer structure. The ITO/Ag/ITO triple-layer structures were prepared using a radio frequency (RF) magnetron sputtering method on a colorless polyimide (CPI) substrate under optimized top and bottom ITO thin film deposition conditions as a function of Ag thickness. The thickness of the CPI substrate was 10 μm. The distance between the CPI substrate and the target was approximately 5 cm, and the substrate holder rotated so that a uniform film morphology was achieved during deposition. The sputtering deposition parameters of the ITO film and Ag thin metal film were base pressure of 3 × 10^−6^ torr, pure Ar flow rate of 50 sccm, and working pressure of 5 × 10^−3^ torr. The RF power of the ITO thin film was 80 W, and for the Ag thin metal film, was 50 W. The thicknesses of the Ag thin metal film were 1.5 nm, 2.9 nm, 5.7 nm, 8.5 nm, 11.4 nm, 14.2 nm, and 17.2 nm with deposition times of 10 s, 20 s, 40 s, 60 s, 80 s, 100 s and 120 s, respectively. The detailed deposition parameters of the ITO thin film and the Ag thin metal film are shown in Table 1. Finally, the ITO/Ag/ITO triple-layer structures were annealed in the air for 1 h at 300 °C.

**Table 1:** Deposition parameters of the ITO/Ag/ITO triple-layer structures

Parameter	Condition		
Target	Bottom ITO	Ag	Top ITO
Substrate	CPI		
Working pressure (torr)	5 × 10^−3^		
Radio frequency power (W)	80	50	80
Substrate temperature (°C)	160	RT	RT
Deposition time (s)	120	10 to 120	120
Argon concentration (sccm)	50		

### 2.2 Analysis of the ITO/Ag/ITO triple-layer structures

Using a UV-Vis spectrometer, the optical transmittances of the ITO/Ag/ITO triple-layer structures were measured at wavelengths between 300 and 1100 nm. The sheet resistances of the ITO/Ag/ITO triple-layer structures were measured using four probe methods. The microstructure of the optimized ITO/Ag/ITO triple-layer structures was examined using high resolution transmission electron microscopy (HR-TEM). The TEM images were obtained from a cross-sectional HR-TEM specimen prepared *via* focus ion beam (FIB) milling. The resistivity (*ρ*), carrier concentration (*n*), and mobility (*μ*) of the ITO/Ag/ITO triple-layer structures were obtained from Hall-effect measurements. The mechanical and temperature reliability of the ITO/Ag/ITO triple-layer structures were investigated using a computer-controlled bending test machine and environmental reliability test system, respectively.

To investigate the feasibility of the ITO/Ag/ITO triple-layer structure as an electrode for capacitive-type TSPs, we fabricated a single-sided ITO (SITO) structure of the TSPs using an ITO/Ag/ITO electrode. The ITO/Ag/ITO electrode was directly deposited, and then patterned by a conventional photolithography and wet-etching process. The flexible printed circuit board (FPCB) bonding with the metal pattern progressed using anisotropic conductive film (ACF), and the FPCB was connected to the touch circuit controller. The touch circuit was designed by HannsTouch Solution Incorporated, in which the touch integrated circuit (IC) was purchased from Synaptics. After the touch sensor was connected to the touch circuit controller, firmware tuning was required. Finally, the plastic cover lens was attached to the touch sensor, and then the fabrication of the TSP was completed.

## 3. Results and discussion

**Fig. 1:**
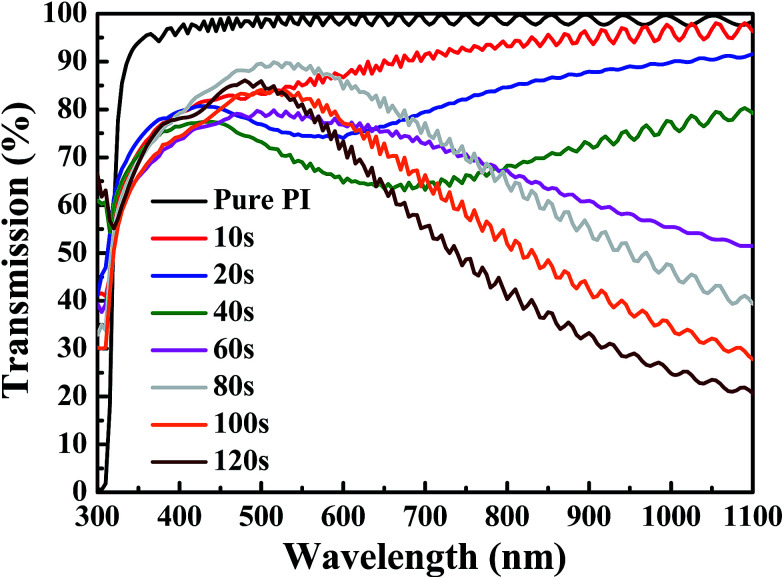
UV-Vis spectra of the flexible ITO/Ag/ITO triple-layer structures as a function of Ag deposition time.

**Fig. 2:**
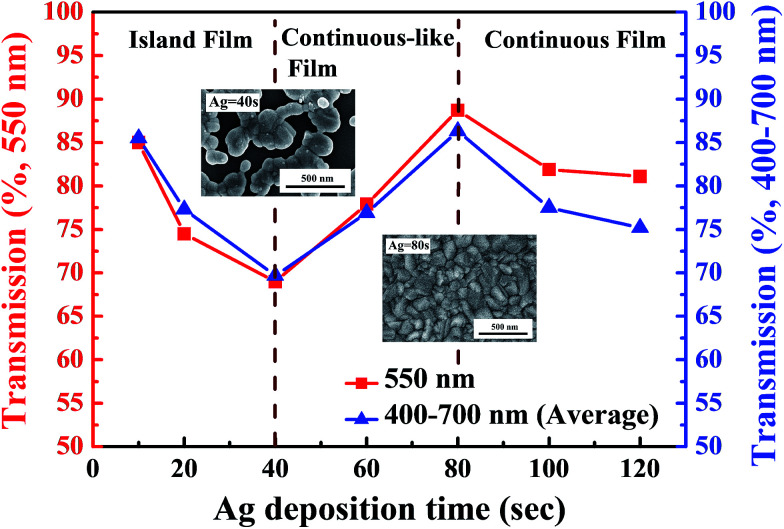
Transmittance of the flexible ITO/Ag/ITO triple-layer structures as a function of Ag deposition time.

In this investigation, a thin metal film of silver (Ag) was inserted between flexible indium tin oxide (ITO) thin films to form ITO/Ag/ITO triple-layer structures. The transmittance spectra of the flexible ITO/Ag/ITO triple-layer structures as a function of Ag deposition time in wavelengths ranging from 300 to 1100 nm are shown in Fig. 1. In Fig. 1, the transmittance of the CPI substrate is 98.1% in the visible region and near-infrared region (NIR) without deposition of the flexible ITO/Ag/ITO triple-layer structures. At Ag deposition times of 10, 20, and 40 s, the transmittance of the flexible ITO/Ag/ITO triple-layer structures shows a sharp increase in the near-ultraviolet (NUV), and then slightly decreases in the visible range, with a subsequent transmittance increase in the NIR. The flexible ITO/Ag/ITO triple-layer structures show a high transmittance in the visible region, with a subsequent slight decrease in the transmittance in the NIR as the Ag deposition time increases from 60 to 80 s. Further increasing the Ag deposition time from 100 to 120 s, the transmittance decreases again in the visible region and rapidly decreases in the NIR. It is obvious that the overall transmittance of the ITO/Ag/ITO triple-layer structures is highly influenced by the Ag deposition time. Fig. 2 shows a transmittance at 550 nm and average transmittance at 400–700 nm of the flexible ITO/Ag/ITO triple-layer structures as a function of Ag deposition time. The transmittance at 550 nm was 85.3%, 74.5%, 69.2%, 77.9%, 88.7%, 81.9%, and 81.1% for Ag deposition times of 10, 20, 40, 60, 80, 100, and 120 s, respectively, as shown in Fig. 2. It was found that the transmittance of the flexible ITO/Ag/ITO triple-layer structures was critically influenced by the thickness of the inserted Ag layer. The increase in Ag deposition times from 10 to 40 s decreased the transmittance at 550 nm and average transmittance at 400–700 nm of the flexible ITO/Ag/ITO triple-layer structures due to severe scattering of light by the separate islands randomly distributed between the ITO layers. Because the Ag islands act as a scattering source of incident light, the flexible ITO/Ag/ITO triple-layer structures are revealed as a bluish color. However, as the Ag deposition time increased from 40–80 s, the Ag islands coalesced to a continuous film that remarkably increased the transmittance and average transmittance in the same wavelength region. The inset in Fig. 2 shows surface SEM images of the ITO/Ag/ITO triple-layer structures that were deposited at the 40 s and 80 s Ag deposition time.

When an optimum Ag thin metal film is embedded between two oxide layers, the resulting oxide/metal/oxide (OMO) triple-layer structure can suppress reflections from the Ag thin metal film and results in a high transmittance in the visible wavelength region.^33–36^ Therefore, the maximum transmittance at 500 nm (88.7%) and 400–700 nm (86.2%) of the flexible ITO/Ag/ITO triple-layer structure was obtained with the Ag deposition time set to 80 s. As the deposition times of the Ag are further increased, the transmittance and average transmittance of the flexible ITO/Ag/ITO triple-layer structures decreases due to the reflection on the thicker Ag metal film. In particular, the optical transmittance in the infrared wavelength region significantly decreased with increasing Ag thin metal film thickness, and this phenomenon was caused by the plasma resonance frequency effect.^37^ Consequently, by insertion of Ag thin metal films with different deposition times between the ITO, the effective plasma resonance frequency of the ITO/Ag/ITO triple-layer structure can be tuned accordingly.

**Fig. 3:**
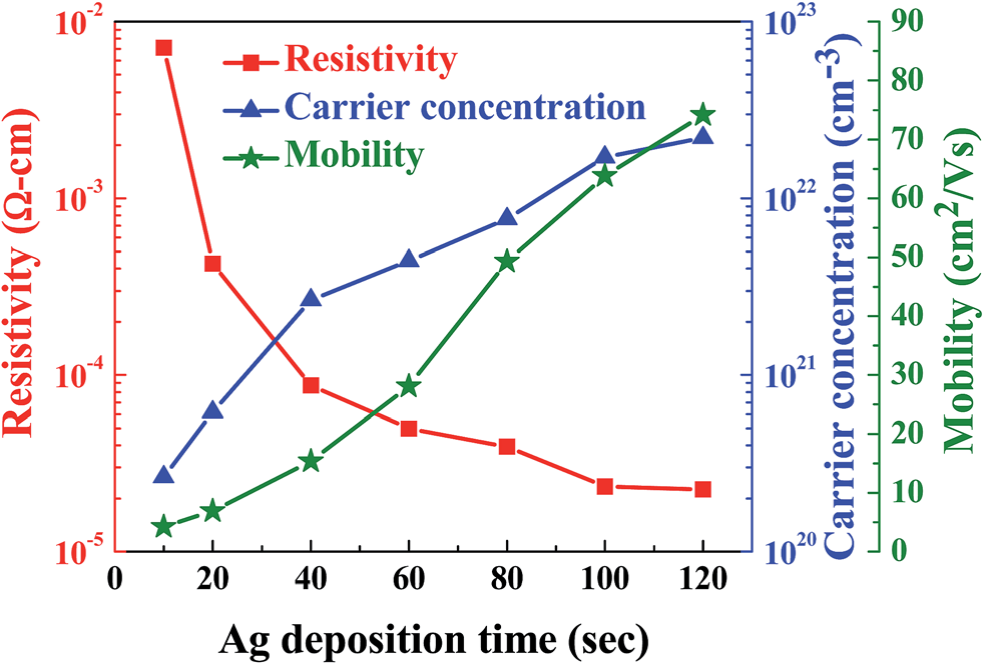
The resistivity (*ρ*), carrier concentration (*n*), and mobility (*μ*) of the flexible ITO/Ag/ITO triple-layer structures as a function of Ag deposition time.

Fig. 3 presents the resistivity (*ρ*), carrier concentration (*n*), and mobility (*μ*) of the flexible ITO/Ag/ITO triple-layer structures as a function of Ag deposition time. The resistivity of the flexible ITO/Ag/ITO triple-layer structures decreased from 9.12 × 10^−4^ to 2.2 × 10^−5^ Ω cm as the Ag deposition time increased from 10 to 120 s. The decrease in resistivity is mainly due to the increases in both carrier concentration and mobility with the increase in Ag deposition time because the resistivity of the flexible ITO/Ag/ITO triple-layer structures is proportional to the reciprocal value of the product of the carrier concentration and the mobility.^38^

**Fig. 4:**
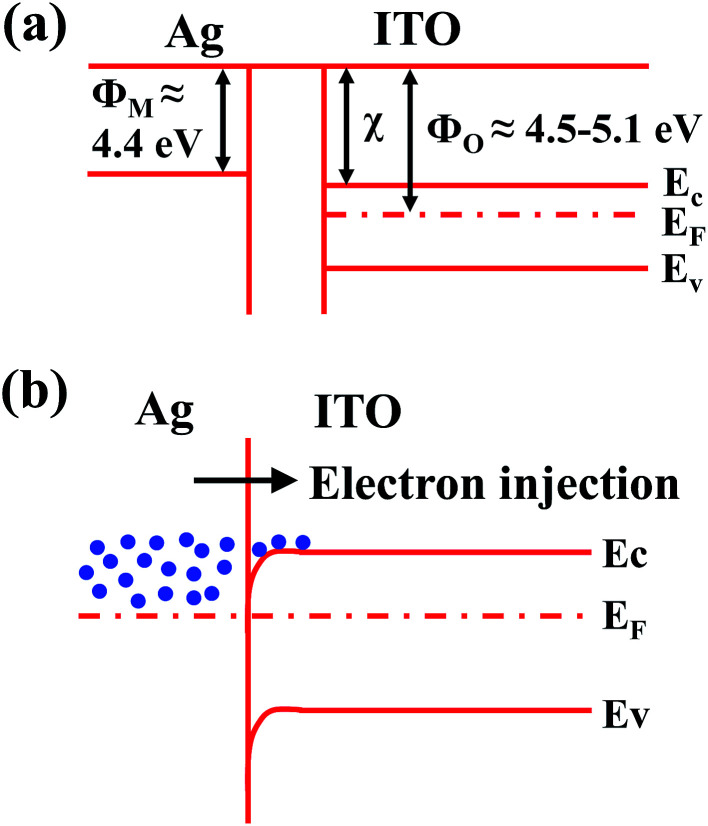
Schematic diagrams of the energy band structures of the ITO and Ag: (a) before contact and (b) after contact.

The carrier concentration increased from 2.65 × 10^20^ cm^−3^ to 2.21 × 10^22^ cm^−3^ as the Ag deposition time increased from 10 to 120 s. As discussed by Alford Klöppel *et al.*, the metal interlayer can act as an electron source for the oxide layer in the TCO/metal/TCO structure.^39^ Therefore, the electron in the Ag layer can be easily injected into the ITO layer due to downward band bending at the contact by the difference in work functions between Ag (*φ*_M_ = 4.4 eV) layer and ITO (*φ*_O_ = 4.5–5.1 eV) in the flexible ITO/Ag/ITO triple-layer structures.^40,41^ A schematic of the energy band diagram of the ITO and Ag prior to and after their contact is shown in Fig. 4(a) and (b), respectively. The mobility of the flexible ITO/Ag/ITO triple-layer structures increased from 4.2 cm^2^ V^−1^ to 74.3 cm^2^ V^−1^ as the Ag deposition time increased. The mobility increase is largely ascribed to the reduction of scattering at the interface regions between the metal and oxide layers, because the interface scattering is considered the main scattering mechanism in the flexible ITO/Ag/ITO triple-layer structure.^42^

**Fig. 5:**
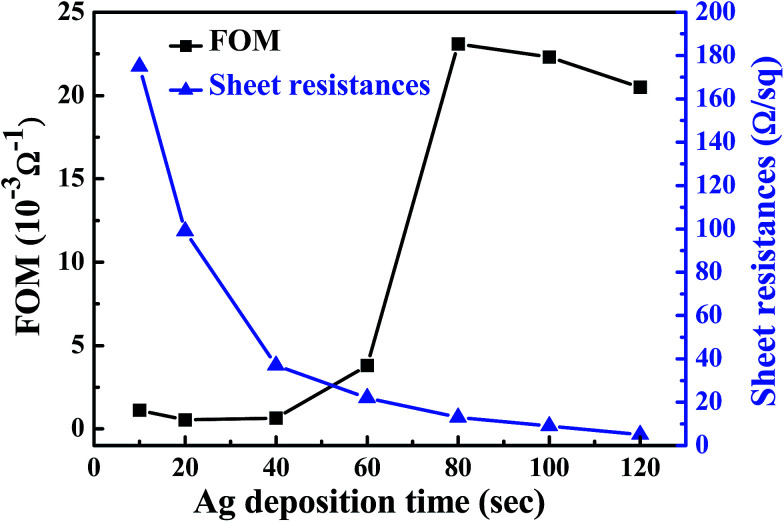
FOM values and sheet resistances of the flexible ITO/Ag/ITO triple-layer structures as a function of Ag deposition time.

Fig. 5 shows the figure of merit (FOM) and sheet resistances of the flexible ITO/Ag/ITO triple-layer structures as a function of Ag deposition time. To obtain the best combination of high transmission and low resistivity, the FOM for the films was calculated using the Haacke equation,^43^1
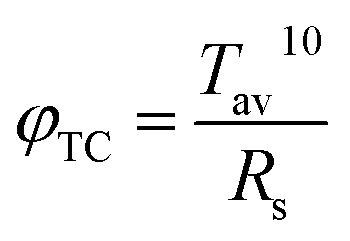
where *T*_av_ is the optical transmittance of the flexible ITO/Ag/ITO triple-layer structures at the 550 nm wavelength, and *R*_s_ is the sheet resistance. *T*_av_ can be estimated using eqn (2):2
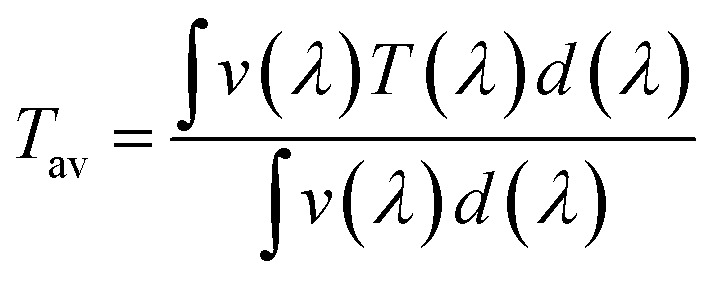
where *T*(*λ*) is the transmittance, and *v*(*λ*) is the photopic luminous efficiency function defining the standard observer for photometry.^44^ In Fig. 5, the maximum value of the FOM at 12.2 × 10^−3^ Ω^−1^ can be observed at 80 s Ag deposition time. The sheet resistance and transmittance at 550 nm were 13 Ω □^−1^ and 88.7%, respectively. From the above results, high optical transmittance and low resistance of the flexible ITO/Ag/ITO triple-layer structure were obtained as the Ag deposition time was set to 80 s [abbreviation as ITO/Ag(80)/ITO].

**Fig. 6:**
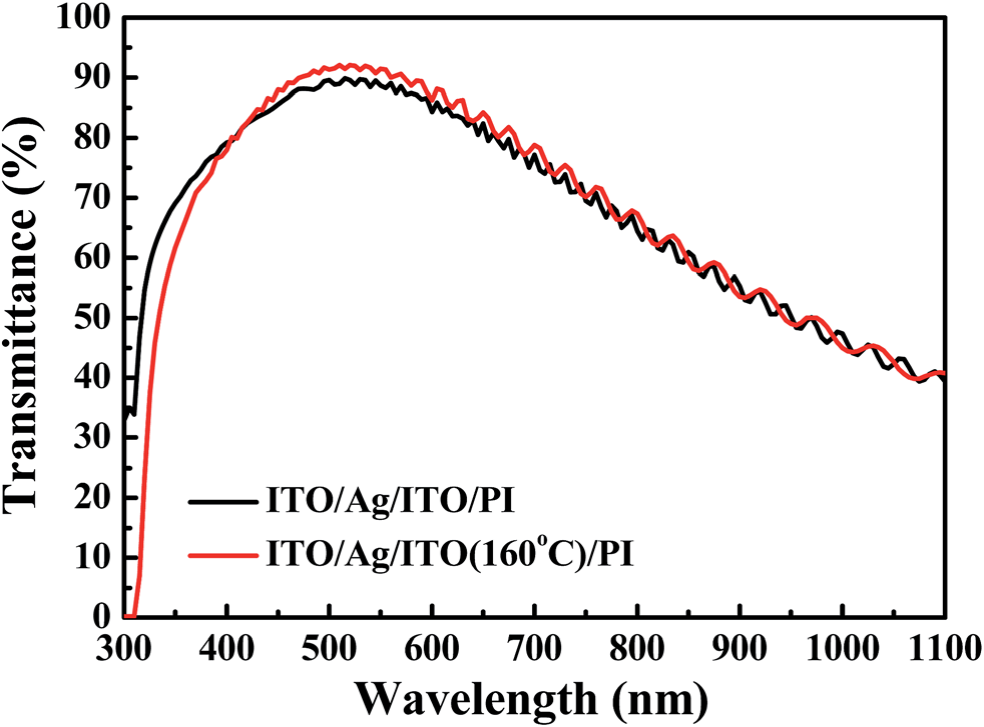
UV-Vis spectra of the flexible ITO/Ag/ITO triple-layer structures with the deposition temperature at 160 °C for the bottom ITO thin film at the 80 s Ag deposition time.

To produce flexible ITO/Ag(80)/ITO triple-layer structures with higher transmittance, the different deposition parameters of the ITO thin film were investigated. Fig. 6 shows the transmittance spectra of the ITO/Ag(80)/ITO triple-layer structures at the deposition temperature of 160 °C for the bottom ITO thin film [abbreviated as ITO/Ag(80)/ITO(160)]. The transmittance at 550 nm and the average transmittance at 400–700 nm of the flexible ITO/Ag(80)/ITO(160) triple-layer structures were 91.4% and 87.5%, respectively. The transmittance and the average transmittance of the flexible ITO/Ag(80)/ITO(160) triple-layer structure are higher than that of the flexible ITO/Ag(80)/ITO triple-layer structure. This was caused by the crystallized ITO thin film, which showed a higher transmittance and lower resistivity than the amorphous film.^45,46^ The sheet resistance of the flexible ITO/Ag(80)/ITO(160) triple-layer structure is 6.4 Ω □^−1^, and it is lower than that of the flexible ITO/Ag(80)/ITO structure. Table 2 gives previously published values for the optical transmittance at 550 nm, sheet resistance, and FOM values of transparent conductive films deposited on flexible substrates. It can be seen that the FOM value obtained in the present work is slightly higher than previously reported values.

**Table 2:** Maximum optical transmittance, minimum sheet resistance, and largest FOM of transparent conductive films reported in the literature

Haacke FOM (10^−3^ Ω^−1^)	Sheet resistance (Ω □^−1^)	Transmission (%; at 550 nm)	Structure	Reference
23.2	13	88.7	ITO/Ag(80)/ITO/CPI	This work
64.57	6.4	91.4	ITO/Ag(80)/ITO(160)/CPI	This work
118.5	1.16	91 (at 600 nm)	ITO/PEI	47
7.87	19.7	83	ITO/PI	48
6.66	8.93	82.4%	ITO/Ag/ITO/PET	49
31.3	4.95	83	Ag NWs/PET	50
13.9	25	90	Cu NWs/PET	51
11.6	30	90	Graphene/PET	52
1.26	85	80	SWNTs/PET	53

**Fig. 7:**
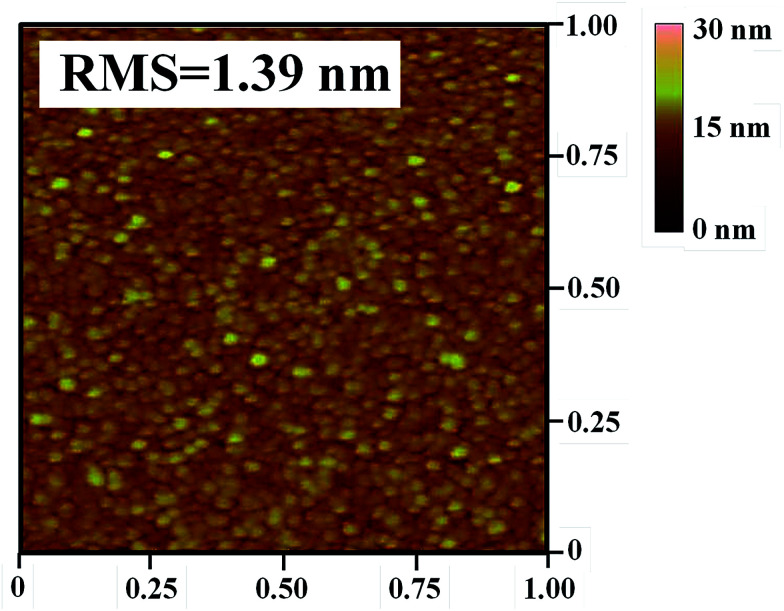
AFM analysis of the flexible ITO/Ag/ITO triple-layer structure with deposition temperature at 160 °C for the bottom ITO thin film at the 80 s Ag deposition time. Scan area is 1 μm × 1 μm.

The surface roughness value of the flexible ITO/Ag(80)/ITO(160) triple-layer structure is very important for future applications. Fig. 7 shows the root mean square (RMS) roughness (1 μm × 1 μm) of the flexible ITO/Ag(80)/ITO(160) triple-layer structure by atomic force microscopy (AFM). The RMS roughness of the flexible ITO/Ag(80)/ITO(160) triple-layer structure is 1.39 nm. The RMS roughness of the top ITO layer is small and demonstrates the amorphous structure and decrease in the surface roughness. Therefore, the flexible ITO/Ag(80)/ITO(160) triple-layer structure is beneficial for further TSP device fabrication.

**Fig. 8:**
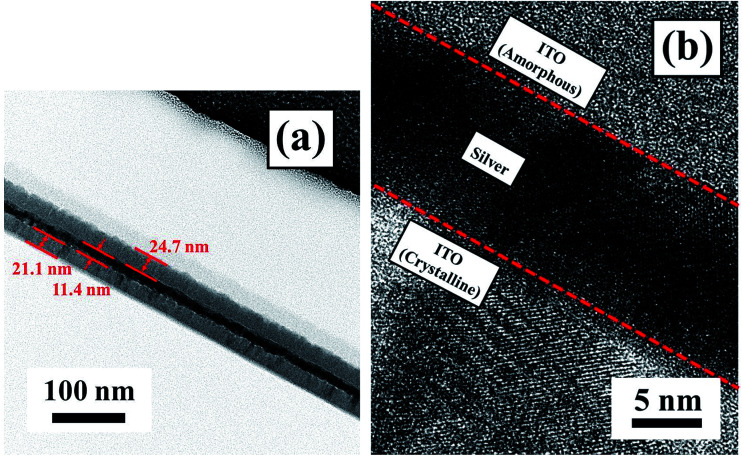
TEM image of the flexible ITO/Ag/ITO triple-layer structure with deposition temperature at 160 °C for the bottom ITO thin film at the 80 s Ag deposition time.

Fig. 8 shows cross-sectional transmission electron microscopy (TEM) images and the high-resolution TEM image of the ITO/Ag(80)/ITO(160) triple-layer structure. The gray and black images clearly show the triple-layer structure consisting of bottom ITO, Ag, and top ITO thin films without interfacial layers, as shown in Fig. 8(a). The thickness of the bottom ITO, Ag, and top ITO thin films are 21.1 nm, 11.4 nm, and 24.7 nm, respectively. In the case of the ITO/Ag/ITO triple-layer structure in Fig. 8(b), the image shows that the bottom ITO thin film has a complete crystallization structure due to its deposition at the substrate temperature of 160 °C. It shows the bottom ITO and Ag layers without an interfacial layer. There are no interface reactions of interfacial oxide layers between the bottom ITO and Ag layers due to the use of a continuous sputtering process without breaking the vacuum. In addition, before deposition of the Ag thin film, the 160 °C substrate temperature would be cooling down to room temperature. Finally, the amorphous structure of the top ITO thin film was obtained at deposition at room temperature. From the above results, it was evident that the crystallization of the ITO thin film enhances the transmittance of the ITO/Ag/ITO triple-layer structure, and the amorphous ITO thin film reduces the surface roughness.

To evaluate the mechanical reliability of the flexible ITO/Ag(80)/ITO(160) triple-layer structure for TSP application, we measured resistance changes (Δ*R*) in the flexible ITO/Ag(80)/ITO(160) triple-layer structure during dynamic outer bending. Table 3 shows the bending reliability analysis of the flexible ITO/Ag(80)/ITO(160) triple-layer structure using a computer-controlled bending test machine with the bending radius set to 3 mm. The critical bending radius of 3 mm was defined by the standard commercial test created by HannsTouch Solution Incorporated. The sheet resistance of the flexible ITO/Ag(80)/ITO(160) triple-layer structure is 6.4 Ω □^−1^. Changes in the sheet resistance of the ITO/Ag(80)/ITO(160) triple-layer structure can be expressed as Δ*R* (%) = (*R*_1_ − *R*_0_)/*R*_0_, where *R*_0_ is the initial resistance and *R*_1_ is the measured resistance after bending. During the 15 000 and 30 000 bending cycles, the Δ*R* values of the flexible ITO/Ag(80)/ITO(160) triple-layer structure were 2.06% and 4.12%, respectively. The dynamic outer bending of the flexible ITO/Ag(80)/ITO(160) triple-layer structure showed less change in sheet resistance after 30 000 bending cycles, demonstrating good flexibility of the ITO/Ag(80)/ITO(160) triple-layer structure. This flexibility of the ITO/Ag(80)/ITO(160) triple-layer structure can be attributed to the ability of the metallic Ag thin interlayer between the ITO layers to withstand high strain, and even the local delamination of or crack formation in the top and bottom ITO thin films.

**Table 3:** Bending reliability analysis and *in situ* measured resistance of the flexible ITO/Ag(80)/ITO(160) triple-layer structure

Bending radius	Δ*R*_15 K_ (%)	Δ*R*_30 K_ (%)
3 mm	2.06%	4.12%

Environmental reliability is a very important test for commercialization of a material. The resistance change (Δ*R*) of the flexible ITO/Ag(80)/ITO(160) triple-layer structure was measured by using the environmental reliability test system. The high and low temperature are set to 80 °C-85% and −40 °C, respectively. The resistance of the flexible ITO/Ag(80)/ITO(160) triple-layer structure is 6.4 Ω □^−1^. During the environmental reliability test of 80 °C-85% and −40 °C, the Δ*R* of the flexible ITO/Ag(80)/ITO(160) triple-layer structure are 2.86% and 0.96% with 240 h as shown in Table 4, respectively. The flexible ITO/Ag(80)/ITO(160) triple-layer structure showed less change in resistance after 80 °C-85% and −40 °C environmental reliability test, demonstrating the good reliability of the flexible ITO/Ag(80)/ITO(160) triple-layer structure.

**Table 4:** Environmental reliability test and *in situ* measured resistance of the flexible ITO/Ag(80)/ITO(160) triple-layer structure

Temperature	Δ*R*_120 h_ (%)	Δ*R*_240 h_ (%)
80 °C-85%	1.71%	2.86%
−40 °C	0.96%	0.96%

**Fig. 9:**
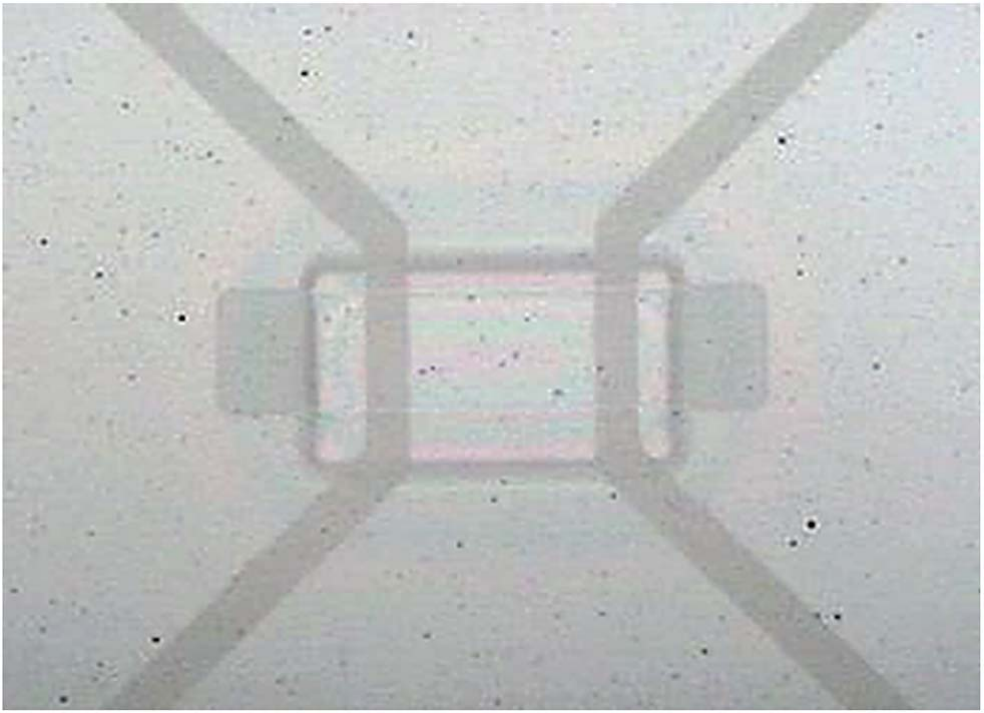
Optical microscope image of the patterned ITO/Ag/ITO triple-layer structure used to fabricate the flexible capacitive-type touch panels.

Fig. 9 shows an optical microscopy (OM) image of the flexible ITO/Ag(80)/ITO(160) triple-layer structure of the fabricated capacitive-type TSPs. To fabricate the flexible capacitive-type TSPs, the ITO/Ag(80)/ITO(160) electrode was patterned by a photolithography and wet etching process. The wet etchant is a phosphoric acid and nitric acid mixture. Using the well patterned ITO/Ag(80)/ITO(160) electrode, flexible capacitive-type TSPs were fabricated.

**Fig. 10:**
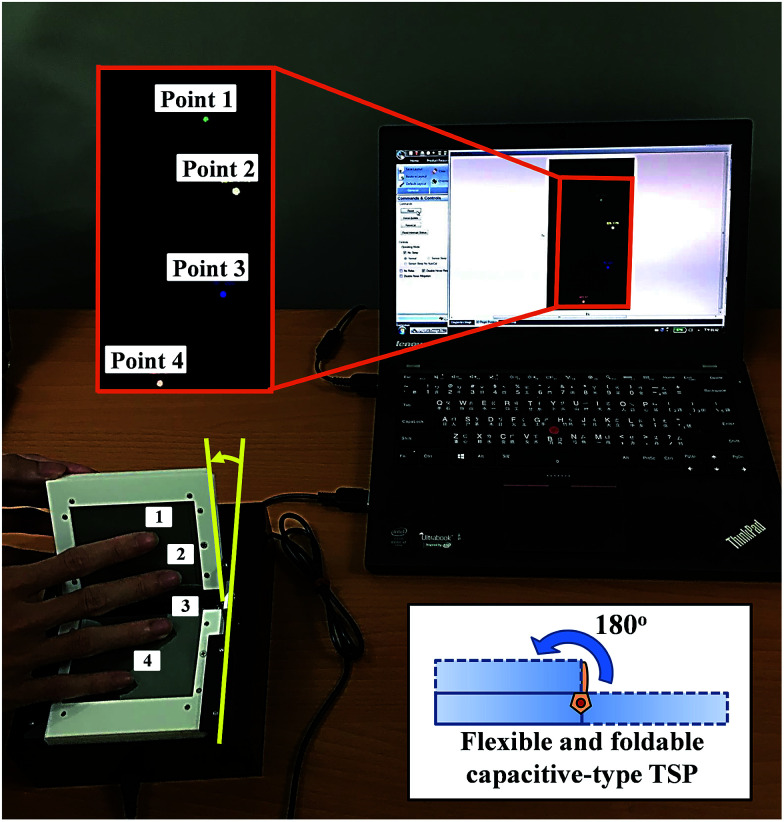
Flexible and foldable capacitive-type TSP fabricated with the ITO/Ag/ITO electrode demonstrates multiple touch point function.

Fig. 10 shows the multiple touch point operation of a flexible capacitive-type TSP fabricated with the ITO/Ag/ITO electrode. The capacitive-type TSP was operated by exact sensing of *X*–*Y* coordinates and characteristics of linearity. Operation of the flexible capacitive-type TSP with the ITO/Ag/ITO electrode indicates that the ITO/Ag/ITO triple-layer structure with low sheet resistance and high optical transmittance is a promising transparent electrode to substitute for the conventional ITO electrode. In this investigation, the maximum bending angle of the flexible capacitive-type TSP was 180°, as shown in Fig. 10. To evaluate the practical usage of the flexible capacitive-type TSP, we lightly touched the TSP, and the four touch points were displayed on a notebook monitor, as shown in Fig. 10. This test verified that this flexible capacitive-type TSP can detect multiple touch points. We are currently in the process of fabricating small-sized capacitive-type TSPs for smartphones and applications combined with organic light emitting diode (OLED) displays.

## 4. Conclusion

An amorphous-indium tin oxide/silver/crystalline-indium tin oxide (a-ITO/Ag/c-ITO) triple-layer structure with low resistivity and high transmittance was studied as a substitute for conventional ITO electrodes for flexible capacitive-type touch screen panels (TSPs). Under optimized conditions, the a-ITO/Ag/c-ITO (21.1 nm/11.4 nm/24.7 nm) triple-layer structure grown on a colorless polyimide (CPI) substrate exhibited a sheet resistance of 6.4 Ω □^−1^, an optical transmittance of 91.4%, a surface roughness of 1.39 nm, a resistance change (Δ*R*) with bending reliability of 4.12%, and Δ*R* of 2.86% and 0.96% with environmental reliability of 80 °C-50%/−40 °C, which is much better than conventional ITO thin film. In this investigation, the flexible and foldable capacitive-type TSP fabricated with a-ITO/Ag/c-ITO electrode demonstrated multiple touch points function.

## Conflicts of interest

There are no conflicts to declare.

## Supplementary Material

## References

[cit1] Formica N., Mantilla-Perez P., Ghosh D. S., Janner D., Chen T. L., Huang M., Garner S., Martorell J., Pruneri V. (2015). An Indium Tin Oxide-Free Polymer Solar Cell on Flexible Glass. ACS Appl. Mater. Interfaces.

[cit2] Pa P. S. (2009). Implementation of a Reuse Process for Liquid Crystal Displays Using an Eccentric-Form Tool. Int. J. Mol. Sci..

[cit3] Shin Y. H., Cho C. K., Kim H. K. (2013). Resistance and transparency tunable Ag-inserted transparent InZnO films for capacitive touch screen panels. Thin Solid Films.

[cit4] Cho S. W., Jeong J. A., Bae J. H., Moon J. M., Choi K. H., Jeong S. W., Park N. J., Kim J. J., Lee S. H., Kang J. W., Yi M. S., Kim H. K. (2008). Highly flexible, transparent, and low resistance indium zinc oxide–Ag–indium zinc oxide multilayer anode on polyethylene terephthalate substrate for flexible organic light-emitting diodes. Thin Solid Films.

[cit5] Singh C., Panda E. (2016). Variation of electrical properties in thickening Al-doped ZnO films: role of defect chemistry. RSC Adv..

[cit6] Maniyara R. A., Mkhitaryan V. K., Chen T. L., Ghosh D. S., Pruneri V. (2016). An antireflection transparent conductor with ultralow optical loss (<2%) and electrical resistance (<6 Ω sq^−1^). Nat. Commun..

[cit7] Bae S., Kim H., Lee Y., Xu X. F., Park J. S., Zheng Y., Balakrishnan J., Lei T., Kim H. R., Song Y. I., Kim Y. J., Kim K. S., Ozyilmaz B., Ahn J. H., Hong B. H., Iijima S. (2010). Nat. Nanotechnol..

[cit8] Fukaya N., Kim D. Y., Kishimoto S., Noda S., Ohno Y. (2014). One-Step Sub-10 μm Patterning of Carbon-Nanotube Thin Films for Transparent Conductor Applications. ACS Nano.

[cit9] Ahn Y., Jeong Y., Lee D., Lee Y. (2015). Copper Nanowire–Graphene Core–Shell Nanostructure for Highly Stable Transparent Conducting Electrodes. ACS Nano.

[cit10] Kim A., Won Y., Woo K., Jeong S., Moon J. (2014). All-Solution-Processed Indium-Free Transparent Composite Electrodes based on Ag Nanowire and Metal Oxide for Thin-Film Solar Cells. Adv. Funct. Mater..

[cit11] Moon H., Won P., Lee J., Ko S. H. (2016). Low-haze, annealing-free, very long Ag nanowire synthesis and its application in a flexible transparent touch panel. Nanotechnology.

[cit12] Suh Y. D., Hong S., Lee J., Lee H., Jung S., Kwon J., Moon H., Won P., Shin J., Yeo J., Ko S. H. (2016). Random nanocrack, assisted metal nanowire bundled network fabrication for a highly flexible and transparent conductor. RSC Adv..

[cit13] Hong S., Yeo J., Lee J., Lee H., Lee P., Lee S. S., Ko S. H. (2015). Selective Laser Direct Patterning of Silver Nanowire Percolation Network Transparent Conductor for Capacitive Touch Panel. J. Nanosci. Nanotechnol..

[cit14] Lee P., Ham J., Lee J., Hong S., Han S., Suh Y. D., Lee S. E., Yeo J. b., Lee S. S., Lee D., Ko S. H. (2014). Highly Stretchable or Transparent Conductor Fabrication by a Hierarchical Multiscale Hybrid Nanocomposite. Adv. Funct. Mater..

[cit15] Lee J., Lee P., Lee H. B., Hong S., Lee I., Yeo J., Lee S. S., Kim T. S., Lee D., Ko S. H. (2013). Room-Temperature Nanosoldering of a Very Long Metal Nanowire Network by Conducting-Polymer-Assisted Joining for a Flexible Touch-Panel Application. Adv. Funct. Mater..

[cit16] Tahar R. B. H., Ban T., Ohya Y., Takahashi Y. (1998). Tin doped indium oxide thin films: electrical properties. J. Appl. Phys..

[cit17] Leterrier Y., Medico L., Demarco F., Manson J. A. E., Betz U., Escola M. F., Olsson M. K., Atamny F. (2004). Mechanical integrity of transparent conductive oxide films for flexible polymer-based displays. Thin Solid Films.

[cit18] Kerkache L., Layadi A., Dogheche E., Remiens D. (2006). Physical properties of RF sputtered ITO thin films and annealing effect. J. Phys. D: Appl. Phys..

[cit19] Choi H. J., Yoon S. G., Lee J. H., Lee J. Y. (2012). Crystallized Indium-Tin Oxide (ITO) Thin Films Grown at Low Temperature onto Flexible Polymer Substrates. ECS J. Solid State Sci. Technol..

[cit20] Sahu D. R., Lin S. Y., Huang J. L. (2006). ZnO/Ag/ZnO multilayer films for the application of a very low resistance transparent electrode. Appl. Surf. Sci..

[cit21] Sahu D. R., Huang J. L. (2006). High quality transparent conductive ZnO/Ag/ZnO multilayer films deposited at room temperature. Thin Solid Films.

[cit22] Kim D. (2010). Low temperature deposition of transparent conducting ITO/Au/ITO films by reactive magnetron sputtering. Appl. Surf. Sci..

[cit23] Jeong J. A., Park Y., Kim H. K. (2010). Comparison of electrical, optical, structural, and interface properties of IZO-Ag-IZO and IZO-Au-IZO multilayer electrodes for organic photovoltaics. J. Appl. Phys..

[cit24] Gong L., Lu J., Ye Z. (2011). Transparent conductive Ga-doped ZnO/Cu multilayers prepared on polymer substrates at room temperature. Sol. Energy Mater. Sol. Cells.

[cit25] Jeong J. A., Park Y. S., Kim H. K. (2010). Comparison of electrical, optical, structural, and interface properties of IZO-Ag-IZO and IZO-Au-IZO multilayer electrodes for organic photovoltaics. J. Appl. Phys..

[cit26] Sahu D. R., Huang J. L. (2006). Dependence of film thickness on the electrical and optical properties of ZnO–Cu–ZnO multilayers. Appl. Surf. Sci..

[cit27] Jung Y. S., Park Y. S., Kim K. H., Lee W. J. (2013). Properties of AZO/Ag/AZO multilayer thin film deposited on polyethersulfone substrate. Transactions on Electrical and Electronic Materials.

[cit28] Park J. H., Choi Y. Y., Kim H. K., Lee H. H. (2010). The effects of rapid thermal annealing on the electrical, optical, and structural properties of Nb:TiO_2_ multilayer electrodes with an inserted nanoscale Ag layer for organic solar cells. J. Appl. Phys..

[cit29] Choi Y. Y., Choi K. H., Lee H., Lee H., Kang J. W., Kim H. K. (2011). Nano-sized Ag-inserted amorphous ZnSnO_3_ multilayer electrodes for cost-efficient inverted organic solar cells. Sol. Energy Mater. Sol. Cells.

[cit30] Fan J. C., Bachner F. J., Foley G. H., Zavracky P. M. (1974). Transparent heat-mirror films of TiO_2_/Ag/TiO_2_ for solar energy collection and radiation insulation. Appl. Phys. Lett..

[cit31] Leftheriotis G., Yianoulis P., Patrikios D. (1997). Deposition and optical properties of optimized ZnS/Ag/ZnS thin films for energy saving. Thin Solid Films.

[cit32] Axelevitch A., Gorenstein B., Golan G. (2012). Investigation of optical transmission in thin metal films. Phys. Procedia.

[cit33] Park Y. S., Kim H. K. (2010). Flexible indium zinc oxide/Ag/indium zinc oxide multilayer electrode grown on polyethersulfone substrate by cost-efficient roll-to-roll sputtering for flexible organic photovoltaics. J. Vac. Sci. Technol., A.

[cit34] Choi K. H., Nam H. J., Jeong J. A., Cho S. W., Kim H. K., Kang J. W., Kim D. G., Cho W. J. (2008). Highly flexible and transparent InZnSnO_*x*_/Ag/InZnSnO_*x*_ multilayer electrode for flexible organic light emitting diodes. Appl. Phys. Lett..

[cit35] Jeong J. A., Kim H. K., Yi M. S. (2008). Effect of Ag interlayer on the optical and passivation properties of flexible and transparent Al_2_O_3_/Ag/Al_2_O_3_ multilayer. Appl. Phys. Lett..

[cit36] Jeong J. A., Kim H. K. (2009). Low resistance and highly transparent ITO-Ag-ITO multilayer electrode using surface plasmon resonance of Ag layer for bulk-hetero junction organic solar cells. Sol. Energy Mater. Sol. Cells.

[cit37] Shin Y. H., Cho C. K., Kim H. K. (2013). Resistance and transparency tunable Ag-inserted transparent InZnO films for capacitive touch screen panels. Thin Solid Films.

[cit38] Chin H. S., Chao L. S., Wu C. C. (2016). Crystal, optical, and electrical characteristics of transparent conducting gallium-doped zinc oxide films deposited on flexible polyethylene naphthalate substrates using radio frequency magnetron sputtering. Mater. Res. Bull..

[cit39] Klöppel A., Kriegseis W., Meyer B. K., Scharmann A., Daube C., Stollenwerk J., Trube J. (2000). Dependence of the electrical and optical behaviour of ITO-silver-ITO multilayers on the silver properties. Thin Solid Films.

[cit40] Milnes A. G. and Feucht D. L., Hetero-Junction and Metal–Semiconductor Junctions, Academic, New York, NY, USA, 1972

[cit41] Sugiyama K., Ishii H., Ouchi Y., Seki K. (2000). Dependence of Indium-Tin-Oxide Work Function on Surface Cleaning Method as Studied by Ultraviolet and X-Ray Photoemission Spectroscopies. J. Appl. Phys..

[cit42] Han H., Theodore N. D., Alford T. L. (2008). Improved conductivity and mechanism of carrier transport in zinc oxide with embedded silver layer. J. Appl. Phys..

[cit43] Haacke G. (1976). New figure of merit for transparent conductors. J. Appl. Phys..

[cit44] Driscoll W. G. and Vaughan W., Handbook of Optics, McGraw-Hill, USA, 1978

[cit45] Kerkache L., Layadi A., Dogheche E., Remiens D. (2006). Physical properties of RF sputtered ITO thin films and annealing effect. J. Phys. D: Appl. Phys..

[cit46] Choi H. J., Yoon S. G., Lee J. H., Lee J. Y. (2012). Crystallized Indium-Tin Oxide (ITO) Thin Films Grown at Low Temperature onto Flexible Polymer Substrates. ECS J. Solid State Sci. Technol..

[cit47] Bazargan A. M., Sharif F., Mazinani S., Naderi N. (2017). A high quality ITO/PET electrode for flexible and transparent optoelectronic devices. J. Mater. Sci.: Mater. Electron..

[cit48] Wen Y., Liu H., Yang S., Fan L. (2015). Transparent and conductive indium tin oxide/polyimide films prepared by high-temperature radio-frequency magnetron sputtering. J. Appl. Polym. Sci..

[cit49] Kim T. H., Park S. H., Kim D. H., Nah Y. C., Kim H. K. (2017). Roll-to-roll sputtered ITO/Ag/ITO multilayers for highly transparent and flexible electrochromic applications. Sol. Energy Mater. Sol. Cells.

[cit50] Xu W., Xu Q., Huang Q., Tan R., Shen W., Song W. (2016). Fabrication of Flexible Transparent Conductive Films with Silver Nanowire by Vacuum Filtration and PET Mold Transfer. J. Mater. Sci. Technol..

[cit51] He T., Xie A., Reneker D. H., Zhu Y., Tough A. (2014). High-Performance Transparent Electrode from a Scalable and Transfer-Free Method. ACS Nano.

[cit52] Bae S., Kim H., Lee Y., Xu X., Park J. S., Zheng Y., Balakrishnan J., Lei T., Kim H. R., Song Y. I., Kim Y. J., Kim K. S., zyilmaz B. O., Ahn J. H., Hong B. H., Iijima S. (2010). Roll-to-roll production of 30-inch graphene films for transparent electrodes. Nat. Nanotechnol..

[cit53] Yim J. H., Kim Y. S., Koh K. H., Lee S. (2008). Fabrication of transparent single wall carbon nanotube films with low sheet resistance. J. Vac. Sci. Technol., B: Microelectron. Nanometer Struct..

